# Condition-based opportunistic maintenance strategy for multi-component wind turbines by using stochastic differential equations

**DOI:** 10.1038/s41598-024-51930-x

**Published:** 2024-01-29

**Authors:** Hongsheng Su, Qian Cao, Yuqi Li

**Affiliations:** School of Automation and Electrical Engineering, Lanzhou Jiaotong University, Lanzhou, 730070 China

**Keywords:** Wind energy, Electrical and electronic engineering

## Abstract

The components of wind turbines are complex in structure and the working environment is harsh, which makes wind turbines face problems such as high failure rates and high maintenance costs. In this paper, the stochastic differential equation model has been established for the harsh operating environment of wind turbines, and used Brownian motion to simulate random disturbances; aiming at the problem of high failure rate of wind turbines, based on Weibull distribution, a new model has been established by combining operating time and equipment state to calculate the failure rate; in the analysis of monitoring data, the Higher-Order Moment method and Bayesian method were used to solve the parameters. The opportunity maintenance threshold curve and preventive maintenance threshold curve were obtained by analyzing Time-Based Maintenance and Condition-Based Maintenance. Therefore, the Condition-Based Opportunistic Maintenance strategy was obtained. The effectiveness of the proposed method was finally verified by arithmetic examples.

## Introduction

Nowadays, wind power generation technology has good development space and huge market potential, and it is a clean energy power generation technology. In 2022, 77.6 GW of newly installed wind power capacity had been connected to the grid globally, which made the total installed capacity of wind power reach 906 GW^1^. Wind power technology has developed rapidly. However, due to the fact that wind farms are usually built in mountainous areas with harsh environments and limited early production capacity, the failure rate of wind turbines is high. In addition, wind farms are generally located in remote areas, making maintenance of wind turbines very difficult and expensive^2,3^. Therefore, a reasonable maintenance strategy is needed to solve these problems.

Preventive maintenance refers to the prevention of functional failures of systems through systematic inspection or regular replacement of products. Spinato’s research^4^ on the WMEP database showed that blades, hub, and pitch control systems were some of the important aspects that affect the fault of wind turbines. Tavner^5^ used the historical data of Germany and Denmark collected from the Windstats survey to analyze the reliability of wind turbine components. Echavarria^6^ showed the changes in reliability of some main components with time by using the database of the German “250 MW wind power generation” test project. In wind farms, the traditional preventive maintenance was TBM. Once the inspection interval was determined, it would not change^7–9^. This strategy couldn't describe the operation of the system in real-time and may lead to over-maintenance or under-maintenance^10–12^. In recent years, there have been many people doing preventive maintenance based on the condition of the system^13–15^. CBM is the process of determining whether a system needs to be repaired by judging its condition, without any over-maintenance or under-maintenance. Gao^16^ conducted a comprehensive analysis of the theory, maintenance methods, and strategy models of CBM for complex equipment. Harsh^17^ established an anomaly detection system for wind turbine gearboxes based on the adaptive threshold, which described the change in the state of the gearbox. As far as a single component is concerned, CBM can reflect the operational status of various components of the wind turbine in real time. However, the neglect of cooperation and coordination during component maintenance can easily lead to frequent shutdowns and increased maintenance costs. Therefore, introducing opportunistic maintenance can effectively solve these problems. Zhao^18^ introduced opportunistic maintenance into the preventive maintenance of wind turbines and adopted a deterministic opportunistic maintenance strategy to achieve optimal maintenance, effectively reducing maintenance costs. Shao^19^ expanded the threshold into a dynamic threshold function, which made the description more reasonable. Zhang and Zhao^20,21^ proposed a maintenance strategy based on reliability, which repaired units that entered opportunistic maintenance when their reliability reached the level of preventive maintenance. Opportunistic maintenance not only ensured the reliability of the unit but also enabled effective maintenance, reducing the possibility of over-maintenance and under-maintenance^22–24^. CBOM combined the advantages of CBM and opportunistic maintenance, which not only considered the state of each component but also provided maintenance opportunities for the remaining components when one component failed^25–27^.

Because of the above problems, considering the number of failures and downtime of each component of the wind turbine, this study selected three key components of the wind turbine: gearbox, pitch system, and blades as the objects of study. When establishing the failure rate model, because the traditional Weibull distribution failure model only considered the influence of time on the equipment failure rate, this study introduced the condition index (*CI*) that reflected the equipment condition information and obtained a comprehensive model that considered the influence of time and condition index on the equipment failure rate at the same time. In data analysis and processing, this study used the high-order moment method and Bayesian estimation method to solve the model parameters. On this basis, the stochastic differential equation model was established. Through the SDE model, the TBM, CBM, and CBOM of wind turbines were analyzed respectively, and the principles and characteristics of these three strategies were explained. Finally, the effectiveness of the CBOM strategy was verified by an example, and it was compared from the time point of view, which showed that CBOM could ensure the stable operation of the system.

## Device state modeling

### Degradation model

The performance of wind turbines is greatly affected by the environment during operation, and wind farms are generally located in remote areas, which makes the degradation process of wind turbines very unstable and prone to random failures. Therefore, it is particularly important to analyze the degradation process of wind turbines.

Definition, *x*(*t*) represented the state of the device at time *t*, when *x*(*t*) = 1, the device was completely undamaged and in good working condition at time *t*, and when *x*(*t*) = 0, the device failed completely at time *t*. The state degradation model of the device was constructed as follows:

1 $${\text{d}}x(t) = f(x(t),t){\text{d}}t + \sigma (x(t),t){\text{d}}B(t)$$

where *f*(*x*(*t*), *t*) represented the decline of the wind turbine itself; *σ*(*x*(*t*), *t*) was the fluctuation rate of the equipment state; *B*(*t*) was Brownian motion, which represented the influence of the external environment on the device state, if *γ* obeyed normal distribution, i.e., *γ* ~ *N*(0,1), then d*B*(*t*) = *γ* $\sqrt {{\text{d}}t}$.

In Eq. (1), For any *T* and *N*, all |*x*(*t*)|≤ *N*, |*y*(*t*)|≤ *N*, and 0 ≤ *t* ≤ *T*, then there was a constant *K* that only depended on *T* and *N* so that *f*(*x*(*t*), *t*) and *σ*(*x*(*t*), *t*) satisfied the following:

2 $$\left| {f\left( {x(t),t} \right) - f\left( {y(t),t} \right)} \right| + \left| {\sigma \left( {x(t),t} \right) - \sigma \left( {y(t),t} \right)} \right| \le K\left| {x(t) - y(t)} \right|$$

3 $$\left| {f(x(t),t)} \right| + \left| {\sigma (x(t),t)} \right| \le K(1 + \left| {x(t)} \right|)$$

and *x*(0) was independent of (*B*(*t*), 0 ≤ *t* ≤ *T*), E*x*^2^(0) < ∞, Then Eq. (1) there was a unique solution *x*(*t*)^28^.

### Failure rate modeling

In the research of preventive maintenance, the Weibull distribution was suitable to represent the operation state of the device. However, the traditional Weibull distribution failure model only considered the influence of time on the failure rate of the device. This paper introduced the condition index that reflects the status information of the device and obtained a comprehensive model that considered both the impact of time and condition index on device failure rate.

The acquisition of the *CI* was as follows: Principal component analysis (PCA) was used to process the characteristic parameters (such as amplitude, temperature, etc.) that affected the device degradation process^29^, and the results were mapped between [0,1] through dispersion standardization, i.e., *CI*(*t*)^30^.

4 $$CI(t) = 1 - \frac{{z_{i} - \min \left\{ z \right\}}}{{\max \left\{ z \right\} - \min \left\{ z \right\}}}$$

where, *z* represented the data when the characteristic parameters were reduced to one dimension, *CI*(*t*) = 1 meant that the device was completely healthy, and *CI*(*t*) = 0 meant that the device completely failed.

Assuming *T*(*CI*) satisfied the exponential relationship of Eq. (5).

5 $$T(CI) = a \cdot e^{b \cdot CI} + c$$

where, *a*, *b*, and *c* were parameters, and *CI* was a condition index.

There were initial conditions:

6 $$CI = 1{ , }T = T_{1}$$

7 $$CI = 0{ , }T = 0$$

**Figure 1:**
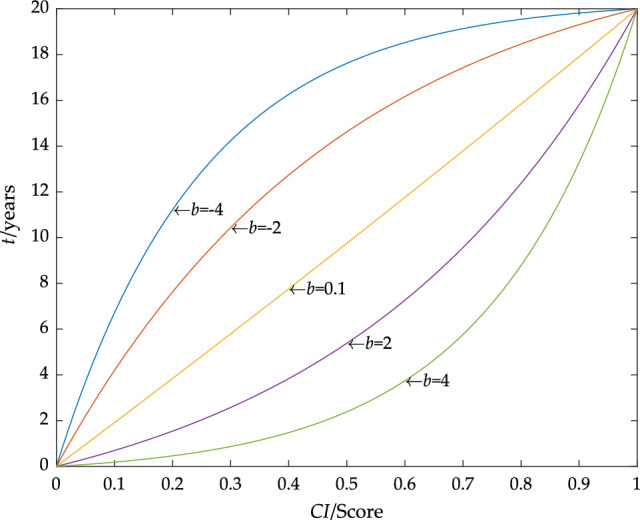
*T*(*CI*, *b*) curves correspond to different *b* values.

The initial conditions of Eq. (5) were given by Eqs. (6) and (7), and it could be concluded that:

8 $$T\left( {CI,b} \right) = T_{1} \cdot \frac{{e^{b \cdot CI} - 1}}{{e^{b} - 1}}$$

where *b* was the parameter to be fitted, *T*_1_ was the factory design life of the device, and *T*_1_ was generally 20 years. The curve of *T*(*CI*, *b*) is shown in Fig. 1.

It can be seen from Fig. 1 that the significance of parameter *b* is that it has established the relationship between operating life, the condition index, and failure rate. When *b* takes different values, the *T*(*CI*, *b*) curve can reflect the characteristics of the convex function, the concave function, and the linear function respectively. In this paper, the *b* value was obtained by fitting the sample data, which could avoid errors caused by differences in the condition index calculation.

**Figure 2:**
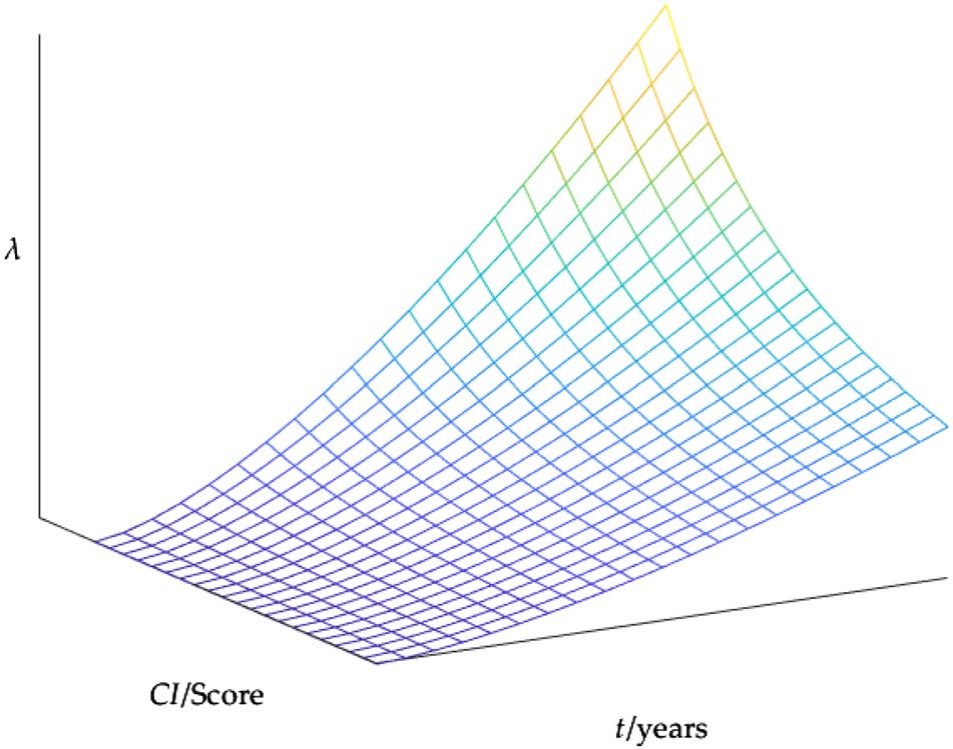
Trend chart of failure rate.

The traditional Weibull distribution formula was as follows:

9 $$\lambda \left( {t,\eta ,\beta } \right) = \frac{\beta }{\eta }\left( {\frac{t}{\eta }} \right)^{\beta - 1}$$

where *β* was the shape parameter of the Weibull distribution; *η* was the curvature parameter of the Weibull distribution, also known as the characteristic life parameter. For the time model based on Weibull distribution, the operating life *T*(*CI*) was used to replace the characteristic life parameter *η*, and the failure rate function was obtained as follows:

10 $$\lambda \left( {t,CI,\beta ,b} \right) = \frac{\beta }{{T_{1} \cdot \frac{{e^{b \cdot CI} - 1}}{{e^{b} - 1}}}}\left( {\frac{t}{{T_{1} \cdot \frac{{e^{b \cdot CI} - 1}}{{e^{b} - 1}}}}} \right)^{\beta - 1}$$

where *β* and *b* were the parameters to be solved, *t* was the age of service, and *CI* was the condition index. The trend chart of failure rate is shown in Fig. 2.

Through the curve diagram of the failure rate shown in Fig. 2, when the condition index decreases, the failure rate increases; As time increases, the failure rate also tends to increase. Therefore, it conformed to practical laws. So, the expression for *f*(*x*(*t*), *t*) was obtained as follows:

11 $$f(x(t),t) = - \lambda (CI,t) \cdot x(t) = - \frac{\beta }{{T_{1} \cdot \frac{{e^{b \cdot CI} - 1}}{{e^{b} - 1}}}}\left( {\frac{t}{{T_{1} \cdot \frac{{e^{b \cdot CI} - 1}}{{e^{b} - 1}}}}} \right)^{\beta - 1} \cdot x(t)$$

### State volatility model

This paper was aimed at the large-scale components that make up the wind turbine, such as blades and gearbox, which needed to be stopped for maintenance. Randomize the minor maintenance caused by inspection, maintenance, and alarm, so it was only related to the device state and had nothing to do with the operating time. Therefore, the state volatility rate could be set as:

12 $$\sigma (x(t),t) = kx(t)$$

### Higher-order moment method

In the following, the parameters *β*, *b,* and *k* were calculated using the higher-order moment method^31^.

For the solution of parameters, first divided the time period [*t*_0_, *T*] into *N* equal intervals, and recorded the length of each segment as Δ*t*, i.e., *t*_*i*+1_−*t*_*i*_ = Δ*t*, *i* = 0, …, *N*−1. Took one of the paths for research.

13 $$x^{(N)} = (x(t_{0} ),x(t_{1} ),...,x(t_{N} ))$$

Using the Euler–Maruyama method for Eq. (1), it could be obtained that:

14 $$x(t_{i + 1} ) - x(t_{i} ) = f(t_{i} ,x(t_{i} ),b,\beta )\Delta t + \sigma (t_{i} ,x(t_{i} ),k)\left[ {B(t_{i + 1} ) - B(t_{i} )} \right]$$

where, *i* = 0, 1, …, *N*−1. From Eq. (14), it could be obtained that:

15 $$x(t_{i + 1} ) - x(t_{i} )\sim N(\Delta t \cdot f(t,x(t_{i} ),b,\beta ),\Delta t \cdot \sigma^{2} (t,x(t_{i} ),k))$$

where, *N*(·, ·) represented normal distribution. Therefore, it could be obtained that:

16 $$\frac{{x(t_{i + 1} ) - x(t_{i} ) - f(t_{i} ,x(t_{i} ),b,\beta )\Delta t}}{{\sigma (t_{i} ,x(t_{i} ),k)}} = B(t_{i + 1} ) - B(t_{i} )$$

Because:

17 $$\frac{{\left[ {B(t_{i + 1} ) - B(t_{i} )} \right]}}{{\sqrt {\Delta t} }}\sim N(0,1)$$

it could be seen that:

18 $$\frac{{x(t_{i + 1} ) - x(t_{i} ) - f(t_{i} ,x(t_{i} ),b,\beta )\Delta t}}{{\sigma (t_{i} ,x(t_{i} ),k)\sqrt {\Delta t} }}\sim N(0,1)$$

From Eq. (18), it could be obtained that:

19 $$Y_{i} \triangleq \frac{{x(t_{i + 1} ) - x(t_{i} ) - f(t_{i} ,x(t_{i} ),b,\beta )\Delta t}}{{\sigma (t_{i} ,x(t_{i} ),k)\sqrt {\Delta t} }}\sim N(0,1)$$

Because:

20 $$E[Y^{n} ] = \left\{ \begin{gathered} 0 \, (n{\text{ is an odd number}}) \hfill \\ (n - 1)!! \, (n{\text{ is an even number}}) \hfill \\ \end{gathered} \right.$$

it could be seen that:

21 $$\left\{ \begin{gathered} \sum\limits_{i = 0}^{N - 1} {\left\{ {\frac{{x(t_{i + 1} ) - x(t_{i} ) - f(t_{i} ,x(t_{i} ),b,\beta )\Delta t}}{{\sigma (t_{i} ,x(t_{i} ),k)\sqrt {\Delta t} }}} \right\}} = 0 \hfill \\ \sum\limits_{i = 0}^{N - 1} {\left\{ {\frac{{x(t_{i + 1} ) - x(t_{i} ) - f(t_{i} ,x(t_{i} ),b,\beta )\Delta t}}{{\sigma (t_{i} ,x(t_{i} ),k)\sqrt {\Delta t} }}} \right\}}^{2} - 1 = 0 \hfill \\ \, \vdots \hfill \\ \sum\limits_{i = 0}^{N - 1} {\left\{ {\frac{{x(t_{i + 1} ) - x(t_{i} ) - f(t_{i} ,x(t_{i} ),b,\beta )\Delta t}}{{\sigma (t_{i} ,x(t_{i} ),k)\sqrt {\Delta t} }}} \right\}}^{h} - E[Y^{h} ] = 0 \hfill \\ \end{gathered} \right.$$

The estimated values of *β*, *b,* and *k* could be obtained by making the *h* equations in (21) equal to zero.

### Bayesian estimation method

The above method was a point estimation method for the parameters and the following Bayesian estimation method was used to consider the quantification of parameter uncertainty^32–36^.

The following deformation was done:

22 $$q{ = }\left( {T_{1} \frac{{e^{b \cdot CI} - 1}}{{e^{b} - 1}}} \right)^{ - \beta }$$

Then the reliability function was as follows:

23 $$R\left( t \right) = \exp \left( { - qt^{\beta } } \right)$$

According to the Bayesian assumption, the uniform distribution was taken as the prior distribution of *β*.

24 $$\pi \left( \beta \right) = \frac{1}{{\beta_{2} - \beta_{1} }} \, \left( {\beta_{1} \le \beta \le \beta_{2} } \right)$$

where the values of *β*_*1*_ and *β*_*2*_ were determined based on engineering experience. Under the given conditions, the conjugate prior distribution Ga(*y*,*z*) was obtained for *q*.

25 $$\pi \left( {q\left| \beta \right.} \right) = \frac{{z^{y} }}{\Gamma \left( y \right)}q^{y - 1} e^{ - z\beta } \, \left( {q > 0,0 < y < 1,z > 0} \right)$$

where *y* and *z* were determined using two quantiles, which could be determined from a priori information and historical information. In this paper, using the upper and lower quartiles *q*_*U*_ and *q*_*L*_, *y* and *z* satisfied the following system of equations:

26 $$\left\{ \begin{gathered} \int_{0}^{{q_{L} }} {\frac{{z^{y} }}{\Gamma \left( y \right)}} q^{y - 1} e^{ - zq} {\text{d}}q = 0.25 \hfill \\ \int_{{q_{U} }}^{\infty } {\frac{{z^{y} }}{\Gamma \left( y \right)}} q^{y - 1} e^{ - zq} {\text{d}}q = 0.25 \hfill \\ \end{gathered} \right.$$

Thus, the joint prior distribution density of *q* and *β* was obtained:

27 $$\pi \left( {q,\beta } \right) = \pi \left( \beta \right)\pi \left( {q\left| \beta \right.} \right) = \frac{1}{{\beta_{2} - \beta_{1} }} \cdot \frac{{z^{y} }}{\Gamma \left( y \right)}q^{y - 1} e^{ - zq} \, \left( {\beta_{1} \le \beta \le \beta_{2} ,q > 0} \right)$$

The *n* samples were taken from the sample and divided into *k* groups, and the number of samples in each group was *n*_*1*_, *n*_*2*_, …, *n*_*k*_, and *n*_*1*_ + *n*_*2*_ +$\cdots$  + *n*_*k*_ = *n*, each group of samples was independently carried out a timed cut-off test, cut-off time was *t*_*1*_, *t*_*2*_, …, *t*_*k*_, and none of the results failed, in which case the corresponding likelihood functions were as follows:

28 $$L\left( {0\left| {q,\beta } \right.} \right) = \exp \left\{ { - \sum\limits_{i = 1}^{k} {n_{i} t_{i}^{\beta } q} } \right\}$$

According to Eqs. (27) and (28) and combined with Bayes’ formula, the joint posterior distribution density of *q* and *β* was obtained:

29 $$\begin{gathered} \pi \left( {q,\beta \left| 0 \right.} \right) = \frac{{\pi \left( {q,\beta } \right)L\left( {0\left| {q,\beta } \right.} \right)}}{{\int\limits_{\beta } {\int\limits_{q} {\pi \left( {q,\beta } \right)L\left( {0\left| {q,\beta } \right.} \right){\text{d}}q{\text{d}}\beta } } }} = \frac{{\frac{1}{{\beta_{2} - \beta_{1} }} \cdot \frac{{z^{y} }}{\Gamma \left( y \right)}q^{y - 1} e^{ - zq} \cdot \exp \left\{ { - \sum\limits_{i = 1}^{k} {n_{i} t_{i}^{\beta } q} } \right\}}}{{\int\limits_{\beta } {\int\limits_{q} {\frac{1}{{\beta_{2} - \beta_{1} }} \cdot \frac{{z^{y} }}{\Gamma \left( y \right)}q^{y - 1} e^{ - zq} \cdot \exp \left\{ { - \sum\limits_{i = 1}^{k} {n_{i} t_{i}^{\beta } q} } \right\}{\text{d}}q{\text{d}}\beta } } }} \\ = \frac{{q^{y - 1} \exp \left\{ { - \left( {z + \sum\limits_{i = 1}^{k} {n_{i} t_{i}^{\beta } } } \right)q} \right\}}}{{\int_{{\beta_{1} }}^{{\beta_{2} }} {\int_{0}^{\infty } {\exp \left\{ { - \left( {z + \sum\limits_{i = 1}^{k} {n_{i} t_{i}^{\beta } } } \right)q} \right\}{\text{d}}q{\text{d}}\beta } } }} = \frac{{q^{y - 1} \exp \left\{ { - \left( {z + \sum\limits_{i = 1}^{k} {n_{i} t_{i}^{\beta } } } \right)q} \right\}}}{{\int_{{\beta_{1} }}^{{\beta_{2} }} {\frac{\Gamma \left( y \right)}{{\left( {z + \sum\limits_{i = 1}^{k} {n_{i} t_{i}^{\beta } } } \right)^{y} }}{\text{d}}\beta } }} \\ \end{gathered}$$

The marginal distribution density of *β* was obtained from the joint distribution density of *q* and *β*.

30 $$\begin{gathered} f\left( {\beta \left| 0 \right.} \right) = \int_{0}^{\infty } {\pi \left( {q,\beta \left| 0 \right.} \right)} {\text{d}}q = \frac{{\int_{0}^{\infty } {q^{y - 1} \exp \left\{ { - \left( {z + \sum\limits_{i = 1}^{k} {n_{i} t_{i}^{\beta } } } \right)q} \right\}{\text{d}}q} }}{{\int_{{\beta_{1} }}^{{\beta_{2} }} {\frac{\Gamma \left( y \right)}{{\left( {z + \sum\limits_{i = 1}^{k} {n_{i} t_{i}^{\beta } } } \right)^{a} }}{\text{d}}\beta } }} = \frac{{\frac{\Gamma \left( y \right)}{{\left( {z + \sum\limits_{i = 1}^{k} {n_{i} t_{i}^{\beta } } } \right)^{y} }}}}{{\int_{{\beta_{1} }}^{{\beta_{2} }} {\frac{\Gamma \left( y \right)}{{\left( {z + \sum\limits_{i = 1}^{k} {n_{i} t_{i}^{\beta } } } \right)^{y} }}{\text{d}}\beta } }} \\ = \frac{{\frac{1}{{\left( {z + \sum\limits_{i = 1}^{k} {n_{i} t_{i}^{\beta } } } \right)^{y} }}}}{{\int_{{\beta_{1} }}^{{\beta_{2} }} {\frac{1}{{\left( {z + \sum\limits_{i = 1}^{k} {n_{i} t_{i}^{\beta } } } \right)^{y} }}{\text{d}}\beta } }} \\ \end{gathered}$$

#### The one-sided confidence interval for β

For a given *α* (0<*α*<1), *β*_*L*_ solved by the following equation

31 $$\int_{{\beta_{L} }}^{{\beta_{2} }} {f\left( {\beta \left| 0 \right.} \right)} {\text{d}}\beta = 1 - \alpha$$

Therefore, Therefore, the one-sided lower confidence limit for a confidence level of 1-*α* for *β* was [*β*_*L*_, *β*_*2*_]. Similarly, the one-sided upper confidence limit for a confidence level of 1-*α* for *β* was [*β*_*1*_, *β*_*U*_], where, *β*_*U*_ was given by the following equation:

32 $$\int_{{\beta_{1} }}^{{\beta_{U} }} {f\left( {\beta \left| 0 \right.} \right)} {\text{d}}\beta = 1 - \alpha$$

#### The shortest confidence interval for β

The following system of equations was obtained through the density of the marginal distribution of *β*.

33 $$\left\{ \begin{gathered} f\left( {\beta_{L} } \right) = f\left( {\beta_{U} } \right) \hfill \\ \int_{{\beta_{L} }}^{{\beta_{U} }} {f\left( {\beta \left| 0 \right.} \right)} {\text{d}}\beta = 1 - \alpha \hfill \\ \end{gathered} \right.$$

Therefore, the shortest confidence interval for a confidence level of 1-*α* for *β* was [*β*_*L*_, *β*_*U*_].

#### The one-sided confidence interval for b

The joint distribution density of *b* and *β* could be obtained from Eqs. (22) and (29).

34 $$\begin{gathered} \pi \left( {b,\beta \left| 0 \right.} \right) = \frac{{\left( {T_{1} \frac{{e^{b \cdot CI} - 1}}{{e^{b} - 1}}} \right)^{{ - \beta }{\left( {y - 1} \right)}} \exp \left\{ { - \left( {z + \sum\limits_{i = 1}^{k} {n_{i} t_{i}^{\beta } } } \right)\left( {T_{1} \frac{{e^{b \cdot CI} - 1}}{{e^{b} - 1}}} \right)^{ - \beta } } \right\}}}{{\int_{{\beta_{1} }}^{{\beta_{2} }} {\frac{\Gamma \left( y \right)}{{\left( {z + \sum\limits_{i = 1}^{k} {n_{i} t_{i}^{\beta } } } \right)^{y} }}{\text{d}}\beta } }} \cdot \left| {\frac{{\ln \left( {T_{1} \frac{{e^{b \cdot CI} - 1}}{{e^{b} - 1}}} \right)}}{{\left( {T_{1} \frac{{e^{b \cdot CI} - 1}}{{e^{b} - 1}}} \right)^{ - \beta } }}} \right| \\ = \frac{{\left( {T_{1} \frac{{e^{b \cdot CI} - 1}}{{e^{b} - 1}}} \right)^{ - \beta y} \exp \left\{ { - \left( {z + \sum\limits_{i = 1}^{k} {n_{i} t_{i}^{\beta } } } \right)\left( {T_{1} \frac{{e^{b \cdot CI} - 1}}{{e^{b} - 1}}} \right)^{ - \beta } } \right\}}}{{\int_{{\beta_{1} }}^{{\beta_{2} }} {\frac{\Gamma \left( y \right)}{{\left( {z + \sum\limits_{i = 1}^{k} {n_{i} t_{i}^{\beta } } } \right)^{y} }}{\text{d}}\beta } }} \cdot \left| {\ln \left( {T_{1} \frac{{e^{b \cdot CI} - 1}}{{e^{b} - 1}}} \right)} \right| \\ \end{gathered}$$

Therefore, the marginal distribution density of *b* is obtained:

35 $$f\left( {b\left| 0 \right.} \right) = \int_{{\beta_{1} }}^{{\beta_{2} }} {\pi \left( {b,\beta \left| 0 \right.} \right)} {\text{d}}\beta$$

For a given α (0 < α < 1), the lower confidence limit of *b* satisfied Eq. (36)

36 $$\int_{{b_{L} }}^{\infty } {f\left( {b\left| 0 \right.} \right)} {\text{d}}b = 1 - \alpha$$

#### The shortest confidence interval for b

From the density of the marginal distribution of *b*, the following system of equations was obtained:

37 $$\left\{ \begin{gathered} f\left( {b_{L} } \right) = f\left( {b_{U} } \right) \hfill \\ \int_{{b_{L} }}^{{b_{U} }} {f\left( {b\left| 0 \right.} \right){\text{d}}b = 1 - \alpha } \hfill \\ \end{gathered} \right.$$

Therefore, the shortest confidence interval for a confidence level of 1-*α* for *b* was [*b*_*L*_, *b*_*U*_].

## Example analysis

In wind turbines, the gearbox, as a power transmission device, was the key to achieving wind turbine growth. Therefore, the gearbox was taken as an example for analysis. The gearbox had been running under the harsh working conditions of variable speed and variable load for a long time, so the faults that were easy to occur include: unbalanced shaft and over-high oil temperature. Therefore, bearing amplitude and oil temperature were selected as monitoring quantities. The simulation analysis data of this paper used the real operation and maintenance data of a wind farm in a period of time. Table 1 shows the operating life of the same type of gearbox during the monitoring period. Table 2 shows the characteristic parameters of this type of gearbox during the monitoring period.

**Table 1:** Operating life of the same type gearbox.

Number	1	2	3	4	5
Operating life (h)	4855	4732	5064	4906	5146

**Table 2:** Characteristic parameters of this type of gearbox.

Number	Operation Time (h)	Oil temperature (°C)	Amplitude (mm)
1	150	23.9	0.355
2	309	25.0	0.365
3	500	26.3	0.470
4	757	26.5	0.603
5	906	27.1	0.743
6	1014	27.4	0.785
7	1540	31.9	1.573
8	2475	37.0	1.704
9	3864	48.2	3.437
10	4855	60.8	5.335

Because the influence of each characteristic parameter on the device was inconsistent, the PCA method was first used to reduce the dimensionality of the data, and then the condition index of the gearbox was calculated according to Eq. (4).

The state deterioration of the gearbox was modeled as:

38 $${\text{d}}x(t) = - \frac{2.01}{{T_{1} \cdot \frac{{e^{( - 1.2) \cdot CI} - 1}}{{e^{( - 1.2)} - 1}}}}\left( {\frac{t}{{T_{1} \cdot \frac{{e^{( - 1.2) \cdot CI} - 1}}{{e^{( - 1.2)} - 1}}}}} \right)^{1.01} \cdot x(t){\text{d}}t + 0.0013 \cdot x(t){\text{d}}B(t)$$

Table 3 shows the historical failure records of some equipment of wind power plants under the EDP Opendata plan in 2016.

**Table 3:** Historical failure records of some equipment of wind power plants.

Turbine_ID	Component	Timestamp	Remarks
T01	GEARBOX	2016-07-18T02:10:00 + 00:00	Gearbox pump damaged
T06	GENERATOR	2016-07-11T19:48:00 + 00:00	Generator replaced
T06	GENERATOR	2016-07-24T17:01:00 + 00:00	Generator temperature sensor failure
T06	GENERATOR	2016-09-04T08:08:00 + 00:00	High temperature generator error
T06	GENERATOR	2016-10-27T16:26:00 + 00:00	Generator replaced
T06	GENERATOR	2016-10-02T17:08:00 + 00:00	Refrigeration system and temperature sensors in generator replaced
T06	HYDRAULIC_GROUP	2016-04-04T18:53:00 + 00:00	Error in pitch regulation
T07	GENERATOR_BEARING	2016-04-30T12:40:00 + 00:00	High temperature in generator bearing (replaced sensor)
T07	TRANSFORMER	2016-07-10T03:46:00 + 00:00	High temperature transformer
T07	TRANSFORMER	2016-08-23T02:21:00 + 00:00	High temperature transformer. Transformer refrigeration repaired
T09	GEARBOX	2016–10-11T08:06:00 + 00:00	Gearbox repaired
T09	GENERATOR_BEARING	2016-06-07T16:59:00 + 00:00	High temperature generator bearing
T09	GENERATOR_BEARING	2016-08-22T18:25:00 + 00:00	High temperature generator bearing
T09	GENERATOR_BEARING	2016-10-17T09:19:00 + 00:00	Generator bearings replaced
T11	GENERATOR	2016-03-03T19:00:00 + 00:00	Electric circuit error in generator
T11	HYDRAULIC_GROUP	2016-10-17T17:44:00 + 00:00	Hydraulic group error in the brake circuit

**Figure 3:**
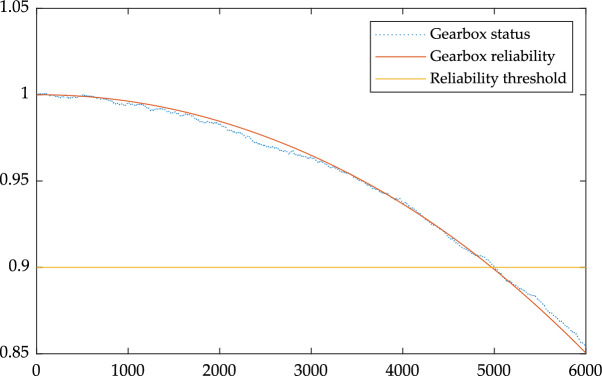
Reliability and state trend diagram of the gearbox.

The reliability curve and state curve of the device were obtained by using MATLAB simulation, and the changing trend of the curves is shown in Fig. 3.

From the curve trend of Fig. 3, it can be concluded that the reliability and status of the gearbox are gradually decreasing, and it can be seen from the decreasing rate that the failure rate increases with time. In actual operation, the longer the operation time, the more likely the gearbox will fail; According to the requirements of reliability, when the reliability of the gearbox is reduced to 0.9, it is necessary to carry out preventive maintenance on gearbox. It can be seen from Fig. 3 that the time when the gearbox reliability reaches the threshold is about 5000 h, which is close to the fault time of 4855 h in Table 1 and close to the failure time of the equipment gearbox in 200 days and 285 days respectively in EDP Opendata 2016, which shows that the model has certain accuracy.

Currently, most researchers use the traditional Weibull proportional risk model in modeling the failure rate. Introducing this model into the SDE model, the state transformation model of the gearbox was obtained as shown below:

39 $${\text{d}}x\left( t \right) = - \frac{\beta }{\eta }\left( {\frac{t}{\eta }} \right)^{\beta - 1} \exp \left( {x\left( t \right)} \right){\text{d}}t + kx\left( t \right){\text{d}}B\left( t \right)$$

**Figure 4:**
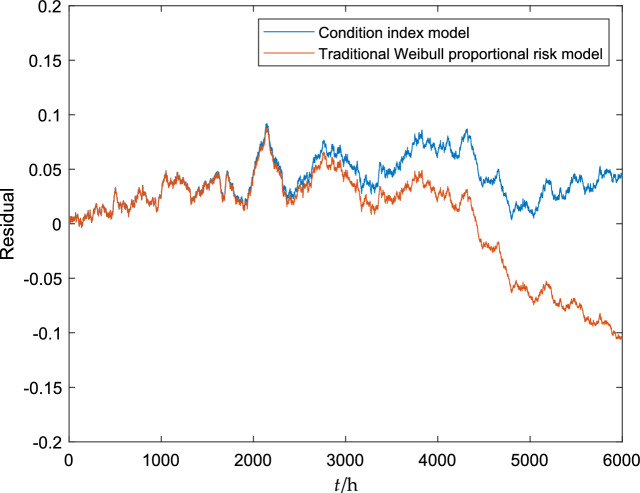
Residual between real state and predicted state of gearbox.

According to Eq. (39), the state transformation model of the gearbox was simulated, and the difference between the real state and the predicted state of the gearbox is shown in Fig. 4.

As can be seen from Fig. 4, the two models are basically similar at the initial stage of equipment operation, which shows that the equipment is relatively less affected by the state at this time. After 3000 h, the state residual of the transformation model using the traditional Weibull proportional risk model has a large mutation, while the residual of the transformation model using the condition index model is relatively stable, which shows that with the increase of time, the equipment state has a greater impact on the equipment operation. It shows that the transformation model based on the condition index can reflect the state of equipment more accurately.

## Strategy Analysis

### TBM strategy

TBM strategy refers to a maintenance strategy with a fixed maintenance cycle. Theoretically speaking, it takes the same time to execute TBM every time. Therefore, the TBM strategy is predictable and can be dealt with in advance. Combined with Eq. (1), it could be seen that under TBM, the state deterioration equation was an ordinary differential equation, so the failure rate *λ* was constant. Because *B*(*t*) was a Brownian motion, the following equation was obtained:

40 $${\text{d}}E(x(t)) = E[\lambda (E(x(t)),t){\text{d}}t] = \lambda E(x(t)){\text{d}}t$$

Through the above analysis, the TBM strategy was an expected strategy in theory. If the execution time of TBM was the same as the actual operation result, then TBM could solve the preventive maintenance problem of the device. Even with certain errors, because the TBM strategy was predictable and fully prepared, the expected effect could be basically achieved. Therefore, the TBM strategy was a scientifically preventive maintenance strategy.

**Figure 5:**
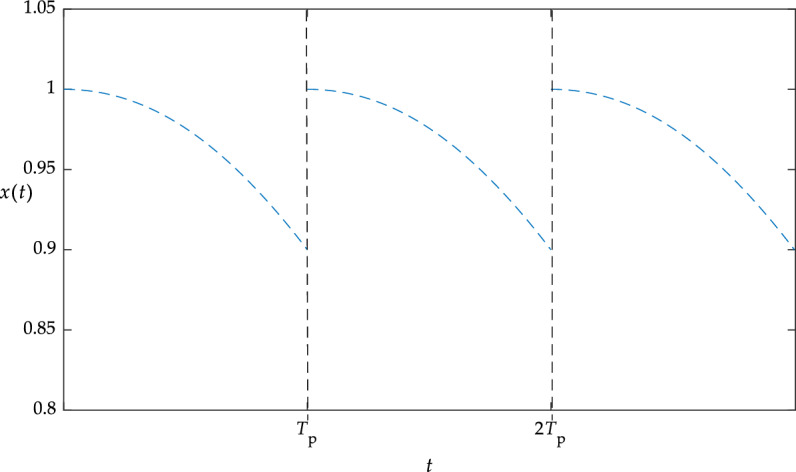
Device status under TBM strategy.

The state of the device under the TBM strategy is shown in Fig. 5, where the device is serviced every *T*_p_ hours.

As can be seen from Fig. 5, the TBM strategy mainly monitors the time and carries out maintenance once it reaches the maintenance time, that is, regular maintenance. TBM can carry out maintenance effectively by arranging maintenance resources and planning downtime in advance. However, TBM does not take into account the actual operation of the device, which is easy to cause under-maintenance and over-maintenance.

### CBM strategy

CBM strategy refers to the strategy of maintenance when the device status reaches the threshold of preventive maintenance. Assuming that the threshold of preventive maintenance was *x*_*p*_, the condition for the device maintenance at time *t*_*m*_ was as follows:

41 $$x(t_{m} ) \le x_{p}$$

It could be seen that *t*_*m*_ was the first time the device status was less than the state threshold *x*_*p*_, which was the downtime of the device. To facilitate CBM analysis, through the modification of Eq. (1), *x*(*t*) could be written as:

42 $$x(t) = x(t_{i} ) + \int_{{t_{i} }}^{t} {\frac{\beta }{{T_{1} \cdot \frac{{e^{b \cdot CI} - 1}}{{e^{b} - 1}}}}\left( {\frac{t}{{T_{1} \cdot \frac{{e^{b \cdot CI} - 1}}{{e^{b} - 1}}}}} \right)^{\beta - 1} x(t){\text{d}}t + \int_{{t_{i} }}^{t} {\sigma (x(t),t){\text{d}}B_{t} } }$$

**Figure 6:**
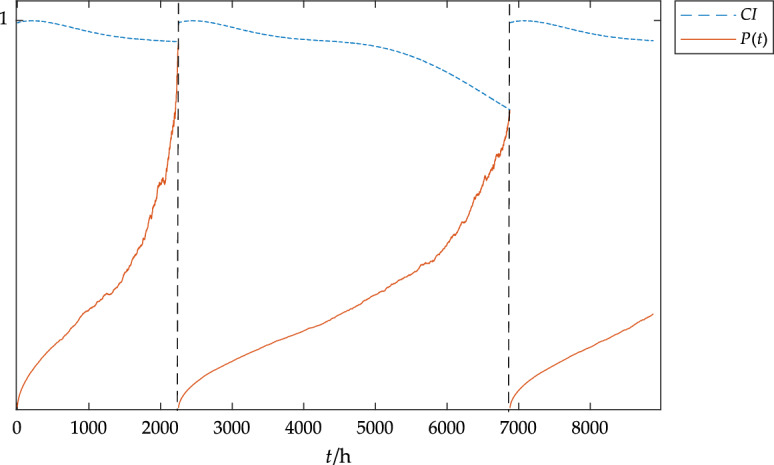
Device status under CBM strategy.

According to Eqs. (41) and (42), the following inequality could be obtained:

43 $$CI \le P(t) = \frac{{\ln \left[ {\frac{{e^{b} - 1}}{{T_{1} }} \cdot \sqrt[\beta ]{{\frac{{\int_{{t_{i} }}^{t} {\beta t^{\beta - 1} x(t){\text{d}}t} }}{{x_{p} - x(t_{i} ) - \int_{{t_{i} }}^{t} {\sigma (x(t),t){\text{d}}B_{t} } }}}} + 1} \right]}}{b}$$

where *P*(*t*) was the threshold function of preventive maintenance. When *CI* ≤ *P*(*t*), according to Eq. (43), it meant that the state had reached the threshold of preventive maintenance and preventive maintenance was needed. The status of devices under the CBM strategy is shown in Fig. 6.

As can be seen from Fig. 6, the CBM strategy monitors the device status, and once the condition index is less than the preventive maintenance threshold, maintenance will be carried out. However, the maintenance time of the device was unpredictable and could not be prepared in advance.

### CBOM strategy

The opportunistic maintenance strategy is applied to multi-component systems, where when one component needs to be repaired, the remaining components also receive a preventive maintenance opportunity. Under this strategy, the simultaneous maintenance of multiple components can significantly improve the availability of the whole device and save total maintenance costs. CBOM combined the advantages of CBM and opportunistic maintenance, utilized real-time monitoring information from various components to reflect the actual operating status of the unit, and judged the opportunistic maintenance time of each component through the opportunistic maintenance threshold and preventive maintenance threshold.

#### Hypothesis 1

The components of the wind turbine could only be repaired after it was shut down.

#### Hypothesis 2

The components of the wind turbine obeyed the Weibull distribution of different parameters and were independent of each other.

#### Hypothesis 3

Opportunistic maintenance adopted incomplete maintenance, and preventive maintenance adopted complete maintenance. Due to the influence of the recovery degree of incomplete maintenance, the components could be repaired twice by opportunistic maintenance at most after complete maintenance.

Based on the derivation of the preventive maintenance threshold in "CBM Strategy" Section, assuming that the preventive maintenance threshold for component 1 was *x*_*p*1_ and the condition-based opportunistic maintenance threshold was *x*_*o*1_, the condition for opportunistic maintenance of component 1 at time *t*_*m*_ was shown in (44).

44 $$x_{p1} \le x_{1} (t_{m} ) \le x_{o1}$$

Let:

45 $$O(t) = \frac{{\ln \left[ {\frac{{e^{b} - 1}}{{T_{1} }} \cdot \sqrt[\beta ]{{\frac{{\int_{{t_{i} }}^{t} {\beta t^{\beta - 1} x(t){\text{d}}t} }}{{x_{o} - x(t_{i} ) - \int_{{t_{i} }}^{t} {\sigma (x(t),t){\text{d}}B_{t} } }}}} + 1} \right]}}{b}$$

46 $$P(t) = \frac{{\ln \left[ {\frac{{e^{b} - 1}}{{T_{1} }} \cdot \sqrt[\beta ]{{\frac{{\int_{{t_{i} }}^{t} {\beta t^{\beta - 1} x(t){\text{d}}t} }}{{x_{p} - x(t_{i} ) - \int_{{t_{i} }}^{t} {\sigma (x(t),t){\text{d}}B_{t} } }}}} + 1} \right]}}{b}$$

**Figure 7:**
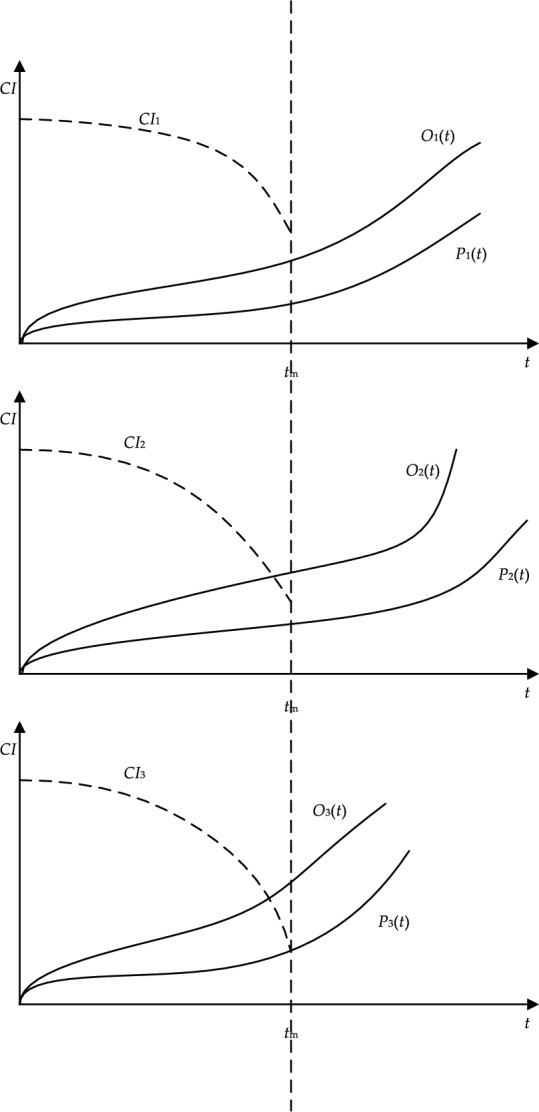
Schematic diagram of CBOM strategy.

Combining Eqs. (43) and (44), the following inequality could be obtained:

47 $$P(t) \le CI \le O(t)$$

where *O*(*t*) was the threshold function of opportunistic maintenance and *P*(*t*) was the threshold function of preventive maintenance. The schematic diagram of the CBOM strategy is shown in Fig. 7.

From Fig. 7, it can be seen that the specific implementation process of the CBOM strategy is as follows: At time *t*_*m*_, the condition index *CI*_3_ of component 3 reaches the preventive maintenance threshold *P*_3_(*t*), and preventive maintenance is carried out; At this point, the condition index of component 2 satisfies *P*_2_(*t*) ≤ *CI*_2_ ≤ *O*_2_(*t*), obtaining an opportunity to repair simultaneously with component 3; Meanwhile, the condition index of component 1 satisfies *CI*_1_ > *O*_1_(*t*), and component 1 does not require maintenance.

### Example analysis

Considering the number of failures and downtime, this paper selected three key components of the wind turbine: blades, pitch system and gearbox for analysis. The simulation analysis data used the real operation and maintenance data of a wind farm in a period of time, the gearbox monitoring data was according to Tables 2 and 4 shows the characteristic parameters of blades during the monitoring period; Table 5 shows the characteristic parameters of pitch system during the monitoring period.

**Table 4:** Characteristic parameters of blades.

Number	Operation time (h)	Waving amplitude (mm)	Swing amplitude (mm)
1	90	0.001	0.002
2	150	0.001	0.002
3	233	0.002	0.003
4	350	0.003	0.005
5	460	0.005	0.006
6	521	0.006	0.008
7	654	0.008	0.011
8	740	0.010	0.015
9	833	0.012	0.017
10	986	0.015	0.02

**Table 5:** Characteristic parameters of pitch system.

Number	Operation time (h)	Motor temperature (°C)
1	705	24.2
2	1500	24.3
3	2208	24.6
4	3090	25.2
5	4460	25.8
6	5471	26.1
7	6637	30.7
8	7540	37.1
9	8703	45.4
10	9640	54.6

**Figure 8:**
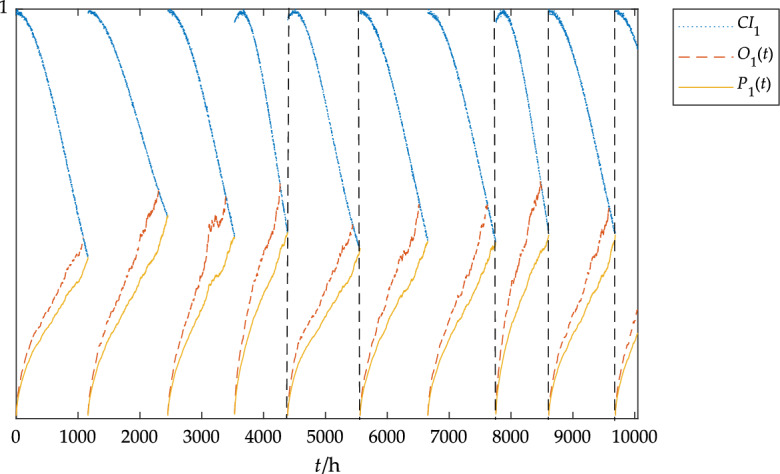
The blades status under the CBOM strategy.

**Figure 9:**
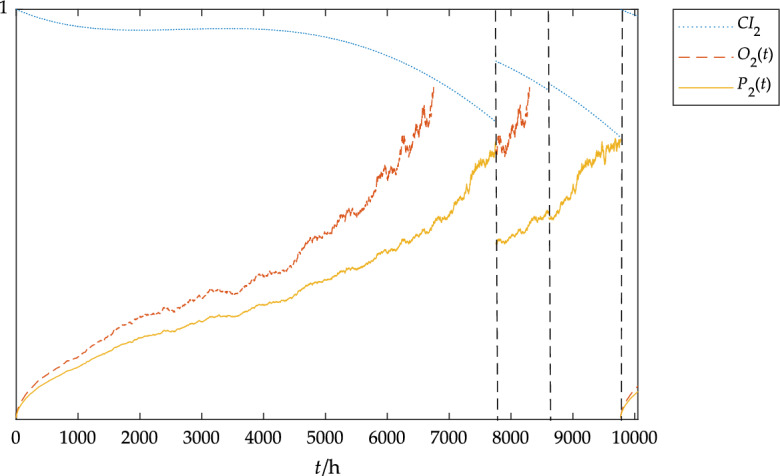
The pitch system status under the CBOM strategy.

**Figure 10:**
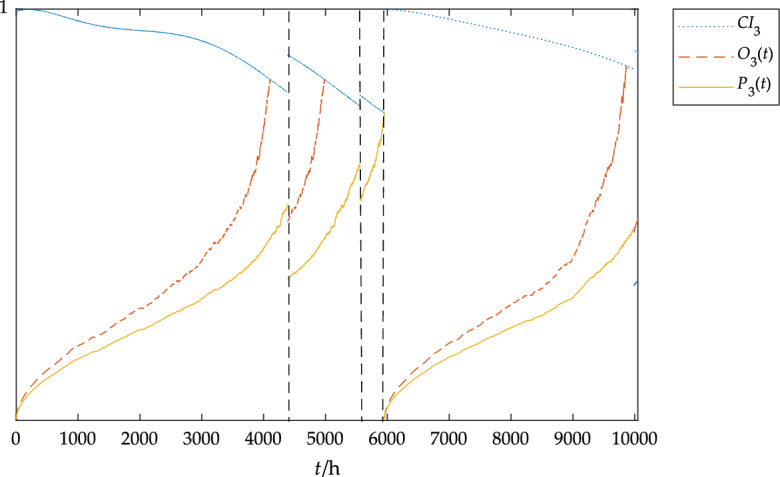
The gearbox status under the CBOM strategy.

The dimension of the data was reduced by the PCA method, and then the condition index of the component was calculated according to Eq. (4). Based on the condition monitoring data in Table 4 and Table 5 combined with the parameter solution method, the parameters of the blade Weibull model could be obtained: *β*_1_ = 2.31, *b*_1_ = − 2.5, *k*_1_ = 0.0023; the parameters of Weibull model of pitch system could be obtained: *β*_2_ = 2.00, *b*_2_ = − 0.3, *k*_2_ = 0.0009; the parameters of Weibull model of gearbox could be obtained: *β*_3_ = 2.01, *b*_3_ = − 1.2, *k*_3_ = 0.0013. The CBOM strategy obtained by MATLAB simulation is shown in Figs. 8, 9 and 10.

In Fig. 8, *CI*_1_ represents the blade condition index, *O*_1_(t) represents the blade opportunistic maintenance threshold curve, and *P*_1_(*t*) represents the blade preventive maintenance threshold curve. As can be seen in Fig. 8, the blades require maintenance approximately every 1,000 h due to the harsh external environment, which provides ample opportunity for repair of other components.

In Fig. 9, *CI*_2_ represents the pitch system condition index, *O*_2_(*t*) represents the pitch system opportunistic maintenance threshold curve, and *P*_2_(*t*) represents the pitch system preventive maintenance threshold curve. As can be seen from Figs. 8 and 9, preventive maintenance is carried out on the blades at 7755 h and 8611 h respectively. At this time, opportunistic maintenance is carried out on the pitch system, which delays the state deterioration process of the pitch system, and finally, preventive maintenance is carried out at about 10000 h.

In Fig. 10, *CI*_3_ represents the gearbox condition index, *O*_3_(*t*) represents the gearbox opportunistic maintenance threshold curve, and *P*_3_(*t*) represents the gearbox preventive maintenance threshold curve. As can be seen from Figs. 8 and 10, preventive maintenance is carried out on the blades at 4390 h and 5560 h respectively. At this time, opportunistic maintenance is carried out on the gearbox, and finally, preventive maintenance is carried out at about 6000 h. Compared with the previous status variation of the gearbox, the life of the gearbox is extended by 16.67% by using opportunistic maintenance, which effectively slows down the state deterioration process of the gearbox.

From the above analysis, it can be concluded that CBOM can delay the deterioration process of device status. Opportunistic maintenance combines the maintenance of multiple components to achieve the purpose of reducing maintenance costs and delaying the deterioration process of the device; CBM solves the hidden problems of opportunistic maintenance and ensures the availability of components. Ultimately, the strategy can ensure the stable operation of the system.

## Conclusions

In this paper, through a profound analysis of the characteristics of the stochastic differential equation and Weibull distribution, combined with the multi-component system, the stochastic differential equation was established to describe the status of each component. On the basis of the CBM strategy, the opportunistic maintenance threshold function and preventive maintenance threshold function were obtained, and the CBOM strategy was established. Through comparative analysis, it had been verified that CBOM could delay the process of device state degradation and ensure stable operation of the system. This strategy is not only suitable for the maintenance of wind turbines but also has certain application prospects for other related fields.

## Data Availability

All data generated or analysed during this study are included in this published article.

## References

[1] Global Wind Energy Council. Available online: Global Wind Report 2022—Global Wind Energy Council (gwec.net) (2022).

[2] Yang LL (2018). Fault diagnosis of wind turbine based on multi-source information fusion-correlation vector machine. Elect. Mach. Control Appl..

[3] Yi Y (2016). Research on early fault diagnosis method of wind turbine based on AR-hankel matrix. Renew. Energy Resour..

[4] Spinato F, Tavner PJ, Van Bussel GJ, Koutoulakos E (2009). Reliability of wind turbine subassemblies. IET Renew. Power Gener..

[5] Tavner PJ, Xiang J, Spinato F (2007). Reliability analysis for wind turbines. Wind Energy.

[6] Echavarria E, Hahn B, van Bussel GJW, Tomiyama T (2008). Reliability of wind turbine technology through time. J. Sol. Energy Eng..

[7] Lu WY, Wang W (2011). Modelling preventive maintenance based on the delay time concept in the context of a case study. Mainten. Reliab..

[8] Christer AH, Wang W (1995). A delay-time-based maintenance model of a multi-component system. Ima J. Manag. Math..

[9] Wang W (2012). An overview of the recent advances in delay-time-based maintenance modelling. Reliab. Eng. Syst. Saf..

[10] Wang XY (2019). Analysis and research on repair interval of equipment. Qual. Reliab..

[11] Takoutsing P (2014). Wind turbine condition monitoring: state-of-the-art review, new trends, and future challenges. Energies.

[12] Pang MT (2013). An optimization method for the calculation of hard-time maintenance interval. Sichuan Ordnance J.

[13] Byon E, Ding Y (2010). Season-dependent condition-based maintenance for a wind turbine using a partially observed Markov decision process. IEEE Trans. Power Syst..

[14] Abdollahzadeh H (2015). Condition based maintenance optimization for multi-state wind power generation systems under periodic inspection. FME Trans..

[15] Hu JW (2021). Condition-based maintenance planning for multi-state systems under time-varying environmental conditions. Comput. Ind. Eng..

[16] Gao, P. Research on Preventive maintenance decision of complex equipment based on reliability analysis, PHD thesis, Tsinghua University, Beijing, 2008.

[17] Dhiman HS (2021). Wind turbine gearbox anomaly detection based on adaptive threshold and twin support vector machines. IEEE Trans. Energy Convert..

[18] Zhao HS, Yan ST, Zhang XT (2014). Research on deterministic opportunity replacement and maintenance strategy of wind turbine. Acta Energy Sin..

[19] Shao, Z.Y. Research on Condition-Opportunity Maintenance Strategy Management System of Wind Turbine, MA thesis, North China Electric Power University, Beijing, 2018.

[20] Zhang LP, Zhao HS (2014). Condition optimization maintenance of fan gearbox based on time delay. Electr. Power.

[21] Zhao HS, Zhang LP (2014). Preventive opportunistic maintenance strategy of wind turbine based on reliability. Proc Chin. Soc. Electr. Eng..

[22] Lu Y (2017). Opportunistic maintenance optimization for offshore wind turbine electrical and electronic system based on rolling horizon approach. J. Renew. Sustain. Energy.

[23] Xie LB (2019). An opportunistic maintenance strategy for offshore wind turbine based on accessibility evaluation. Wind Eng..

[24] Liu GH (2021). Optimum opportunistic maintenance schedule incorporating delay time theory with imperfect maintenance. Reliab. Eng. Syst. Saf..

[25] Koochaki J (2012). Condition based maintenance in the context of opportunistic maintenance. Int. J. Prod. Res..

[26] Jiang AP (2018). A condition-based opportunistic maintenance policy integrated with energy efficiency for two-component parallel systems. J. Ind. Eng. Manag..

[27] Zhang XH (2021). Optimal condition-based opportunistic maintenance and spare parts provisioning for a two-unit system using a state space partitioning approach. Reliab. Eng. Syst. Saf..

[28] Klebaner, F.C. *Introduction to Stochastic Calculus with Applications*, 2rd ed.; The People's Posts and Telecommunications Press: Beijing, China, 2008; pp. 133–134.

[29] Zhao SK (2018). A method for predicting the remaining life of mechanical system based on data-driven and Bayesian theory. J. Mech.Eng..

[30] Li ZE (2021). Health index-based condition assessment and prediction of high-speed shaft bearings of wind turbines. Acta Energy Sin..

[31] Qi, F.T. Parameter Estimation of Stochastic Differential Equations, MA thesis, Shandong University, Qingdao, 2017.

[32] Kampitsis D (2022). A Bayesian condition-based maintenance and monitoring policy with variable sampling intervals. Reliab. Eng. Syst. Saf..

[33] Zhou SR (2023). Fast bayesian inference of reparameterized gamma process with random effects. IEEE Trans. Reliab..

[34] Wang, W.W., et al. Regression analysis of clustered panel count data with additive mean models. *Statistical Papers*, 2023.

[35] Wu SN (2022). Hybrid dynamic Bayesian network method for performance analysis of safety barriers considering multi-maintenance strategies. Eng. Appl. Artif. Intell..

[36] Zhai, Y.M. Bayesian Analysis for Weibull Distribution, MA thesis, Southwest Jiaotong University, Chengdu, 2002.

